# Detection and discrimination of multiple strains of Zika virus by reverse transcription-loop-mediated isothermal amplification

**DOI:** 10.1186/s41182-020-00274-z

**Published:** 2020-10-21

**Authors:** Hiroka Aonuma, Itoe Iizuka-Shiota, Tokio Hoshina, Shigeru Tajima, Fumihiro Kato, Seiji Hori, Masayuki Saijo, Hirotaka Kanuka

**Affiliations:** 1 Department of Tropical Medicine, The Jikei University School of Medicine, Tokyo, Japan; 2 Center for Medical Entomology, The Jikei University School of Medicine, Tokyo, Japan; 3 Present Address: Department of Genetics, University of North Carolina at Chapel Hill, Chapel Hill, USA; 4 Department of Infectious Diseases and Infection Control, The Jikei University School of Medicine, Tokyo, Japan; 5 Department of Virology 1, National Institute of Infectious Diseases, Tokyo, Japan

**Keywords:** Zika fever, Zika virus, RNA, Loop-mediated isothermal amplification, Patient

## Abstract

**Background:**

Monitoring both invasion of Zika virus disease into free countries and circulation in endemic countries is essential to avoid a global pandemic. However, the difficulty lies in detecting Zika virus due to the large variety of mutations in its genomic sequence. To develop a rapid and simple method with high accuracy, reverse transcription-loop-mediated isothermal amplification (RT-LAMP) was adopted for the detection of Zika virus strains derived from several countries.

**Results:**

Common primers for RT-LAMP were designed based on the genomic sequences of two standard Zika strains: African lineage, MR-766, and Asian lineage, PRVABC59. RT-LAMP reactions using a screened primer set, targeting the NS3 region, detected both Zika virus strains. The minimum detectable quantity was 3 × 10^−2^ ng of virus RNA. Measurable lag of reaction times among strains was observed. The RT-LAMP method amplified the target virus sequence from the urine and serum of a patient with a travel history in the Caribbean Islands and also provided a prediction about which lineage of Zika virus strain was present.

**Conclusions:**

The RT-LAMP method using a well-optimized primer set demonstrated high specificity and sensitivity for the detection of Zika virus strains with a variety in genomic RNA sequences. In combination with the simplicity of LAMP reaction in isothermal conditions, the optimized primer set established in this study may facilitate rapid and accurate diagnosis of Zika fever patients with virus strain information.

## Background

Zika virus (ZIKV) is an arbovirus belonging to the family *Flaviviridae*. ZIKV is transmitted by biting of infected *Aedes* mosquitoes mainly in tropical regions in the same manner as dengue, chikungunya, and yellow fever. Zika fever (also known as Zika virus disease) became a globally important medical issue with the declaration of a Public Health Emergency of International Concern by the World Health Organization in February 2016. ZIKV was first identified in 1947 in Uganda, attracting little attention till an association between ZIKV and microcephaly was suspected after a large outbreak in Brazil in 2015 [1, 2]. Most cases of ZIKV infection are thought to be asymptomatic, but some with symptomatic cases showing rash, fever, and arthralgia, and rarely with severe symptomatic Guillain-Barré syndrome. A higher burden of ZIKV on humans occurs particularly in infection during pregnancy, causing microcephaly and other fetal outcomes [3].

Loop-mediated isothermal amplification (LAMP) is a DNA amplification method utilizing DNA polymerase such as *Bst* and OmniAmp®, which is capable of amplification in isothermal conditions with four to six specific primers [4, 5]. LAMP has emerged as an advantageous alternative to PCR-based methodologies, mostly due to its simplicity and utility without specialized equipment. LAMP amplifies a target DNA with high specificity using multiple primers, and it has been used to type single nucleotide polymorphisms in a DNA sequence [6–8]. It has also been widely used for the detection of mosquito-borne pathogens such as parasites and viruses from both human and vector mosquitoes [9–13]. Reverse transcription (RT)-LAMP has been employed to detect genomic RNA of ZIKV [14–17], because recent outbreaks have occurred in resource-limited settings, increasing the need for simple assays suitable for reliable, cost-effective, high-throughput ZIKV diagnosis.

The difficulty in detecting viruses is still apparent due to the high genomic diversity of viruses [18]. Sequencing of viral genomes and phylogenetic analysis have supported spatio-temporal comparisons of genetic variation. Genomic epidemiology approaches have successfully reconstructed the emergence of ZIKV via mapping spread and evolution of the virus in the Americas [19–21]. Recently, the complete genome sequences of two major distinct ZIKVs were determined: MR-766-NIID, an African ZIKV isolate identified from a febrile sentinel monkey in the Zika forest near Entebbe, Uganda, in 1947 [1], and PRVABC59, an American epidemic strain isolated from the blood of a human in Puerto Rico in December 2015 [22]. Phylogenetic analyses using the sequences of the 29 available ZIKV genomes categorized these two strains into major genetic lineages, African (MR-766-NIID) and Asian (PRVABC59) [23].

In this study, the ability of RT-LAMP to detect two standard ZIKV strains, African MR-766-NIID and Asian PRVABC59, with a newly optimized common primer set, was evaluated for their sensitivity and specificity. RT-LAMP using this primer set also detected an unidentified ZIKV strain collected from a Zika fever patient, highlighting the high specificity of RT-LAMP as a noteworthy advantage in diagnosing vector-borne diseases.

## Methods

### Cell culture

ZIKVs used in this study were cultured in either *Aedes albopictus* C6/36 cells (a gift from Dr. Gong Cheng) or African green monkey kidney Vero cells (ATCC) or both. C6/36 cells were cultured in D-MEM, high glucose (Thermo Fisher Scientific Inc.) supplemented with 10% fetal bovine serum (FBS) and penicillin-streptomycin. Vero cells were cultured in E-MEM supplemented with 10% FBS, MEM Non-Essential Amino Acids Solution (Thermo Fisher Scientific Inc.), and penicillin-streptomycin.

### Virus isolation and propagation

Two standard ZIKV strains, MR-766-NIID (African lineage) and PRVABC59 (Asian lineage), were passaged in Vero cells. For virus isolation from a ZIKV-infected patient, both C6/36 and Vero cells were used. Briefly, the patient’s urine was diluted in culture medium and initially inoculated into C6/36 cells at 28 °C. Part of the culture supernatant was then inoculated into fresh C6/36 cells. After several additional passages in viral cell culture using Vero cells at 37 °C in 5% CO_2_ for several days, viral replication was confirmed by observing a cytopathic effect and increased ZIKV RNA load in cell supernatant with a conventional RT-PCR method. Briefly, cDNAs of ZIKV were synthesized using M-MLV reverse transcriptase (Thermo Fisher Scientific Inc.) according to the manufacturer’s instructions. PCR reaction was done using TaKaRa Ex Taq (Takara Bio Inc.) following the manufacturer’s directions with a primer set targeting the partial sequence of the region for envelope (forward: GCTGGDGCRGACACHGGRACT, reverse: RTCYACYGCCATYTGGRCTG) [24]. The target sequence was amplified using the following procedure: 1 cycle at 98 °C for 3 min and 34 cycles at 94 °C for 10 s, at 55 °C for 30 s, and at 72 °C for 30 s. Amplified products were confirmed by gel electrophoresis. The ZIKV isolate was then maintained in Vero cells.

### Patient sample

We analyzed samples from a patient with confirmed ZIKV infection enrolled in a descriptive, observational study conducted at the Jikei University Hospital (Tokyo, Japan). The study protocol was approved by an institutional review board (permission number 28-023 [8266] from the Jikei University School of Medicine). Individuals of all ages and any gender that met the case definition of suspected acute Zika (fever and/or rash, and one or more other symptoms including arthralgia, myalgia, non-purulent conjunctivitis or conjunctival hyperemia, headache, and malaise) were eligible for the study. Informed consent was obtained from every participant. After consent, serum and urine samples were obtained at each visit. In the current study, one patient, who visited the Dominican Republic in 2016 and returned to Japan, with confirmed ZIKV infection in serum and urine was included. ZIKV RNA was detected from this patient in serum using real-time RT-PCR performed at the National Institute of Infectious Diseases in Japan with primer-probe sets described previously [25]. Serum and urine were collected from the patient and stored at − 80 °C for further analysis.

### Viral RNA extraction

The culture supernatant of virus-infected Vero cells was centrifuged at 430×*g* for 15 min at 4 °C to eliminate host cells. Viral RNAs from the supernatant were extracted with High Pure Viral Nucleic Acid Kit (Roche Ltd.) in accordance with the manufacturer’s instructions with slight modifications. Collected RNA (mostly consisting of viral RNA) was serially diluted and provided for further experiments.

For the extraction of viral RNA from the patient, urine and serum samples of the ZIKV-infected patient were thawed on ice. Total RNA was extracted from 250 μl of each sample as follows: each sample was mixed well with 750 μl of TRIzol LS (Thermo Fisher Scientific Inc.) and incubated at room temperature for 5 min. Next, 200 μl of chloroform was added and incubated at room temperature for 15 min. Five hundred microliters of supernatant was collected by centrifugation at 20,400×*g* for 15 min at 4 °C and mixed with 500 μl of isopropanol. RNA was precipitated by centrifugation at 20,400×*g* for 10 min at 4 °C, rinsed with 70% ethanol, and dried. RNA was diluted in 21 μl of RNase-free water. 2.5 μl of each RNA solution was used as a template for the RT-LAMP reaction.

### RT-LAMP reaction

The primer set for RT-LAMP was designed using PrimerExplorer V5 (Fujitsu Ltd.). The RT-LAMP reaction was performed in accordance with the manufacturer’s instructions (Eiken Chemical Co., Ltd.) with slight modifications. Briefly, the reaction was performed in a 12.5 μl reaction mixture (2.5 μl of extracted RNA solution, 0.5 μl of Enzyme Mix, 3.25 μl of primer solution [2.5 pmol of each F3 and B3 primers, 20 pmol of each FIP and BIP primers, 10 pmol of each Loop-F and Loop-B primers], and 6.25 μl of 2× reaction mixture). The reaction mixture was incubated at 62 °C for 60 min using a Loopamp Realtime Turbidimeter (LoopampEXIA; Eiken Chemical Co., Ltd.). The reaction was terminated by incubation at 80 °C for 5 min. Amplified products of the reaction were examined by electrophoresis in 2% agarose gels at 50 V. The gels were stained with ethidium bromide and visualized under UV light at 312 nm.

### Viral sequence analysis

To analyze and compare sequences among ZIKV strains, cDNA of ZIKV derived from an infected patient was synthesized, amplified, and sequenced. Briefly, 1 μg of extracted RNA dissolved in 10.5 μl of RNase-free water was incubated with a reaction mixture (3 μl of 40 μM Random Primers (Thermo Fisher Scientific Inc.), 6 μl of 5× First-Strand Buffer (Thermo Fisher Scientific Inc.), 1.9 μl of 0.1 M DTT (Thermo Fisher Scientific Inc.), and 7.5 μl of dNTP Mixture (Takara Bio Inc.)) at 65 °C for 5 min. 0.6 μl of RNase inhibitor (Promega Co.) and 0.5 μl of M-MLV reverse transcriptase (Thermo Fisher Scientific Inc.) were added to the reaction mixture and incubated at 37 °C for 90 min. Partial cDNA was amplified by PCR using PrimeSTAR Max DNA polymerase (Takara Bio Inc.) with a primer set (forward: TCACAGATCCCTCAAGTATAGC, reverse: CTCTGAAATGTCAGTTGTCACG) or F3 and B3 primers for RT-LAMP (Table 1). Amplified product was purified with a QIAEX II Gel Extraction Kit (QIAGEN) and sequenced directly with each primer used for amplification. Determined sequence was aligned with the sequences of 6 ZIKV strains (from NCBI: PRVABC59, DOM_2016_BB-0076-SER (GenBank: KY014305.2), DOM_2016_BB-0183-SER (GenBank: KY785420.1), DOM_2016_BB-0127-SER (GenBank: KY014303.2), DOM_2016_BB-0208-SER (GenBank: KY014300.2), and DOM_2016_BB-0180-URI (GenBank: KY785476.1)).

**Table 1:** Sequences of primers for RT-LAMP designed in accordance with the nucleotide sequence of ZIKV MR strain

Primer	Sequence (5′–3′)
F3	TTATGACTGCCACACCACC
B3	GCTGAGCTGTATGACCCG
FIP (F1c-F2)	GGCTCTCTCTGGGACTTCCACTGAACCCGTGATGCGTTTCC
BIP (B1c-B2)	TTGGTTCGTTCCAAGCGTGAGATTTCCAGCCTTTGTCAGACA
Loop-F	GTCCATGATTGGTGAGTTAGAGTCA
Loop-B	AACGGAAATGAAATCGCAGCC

## Results

### Design and screening of optimized primer set for ZIKV RT-LAMP

**Fig. 1:**
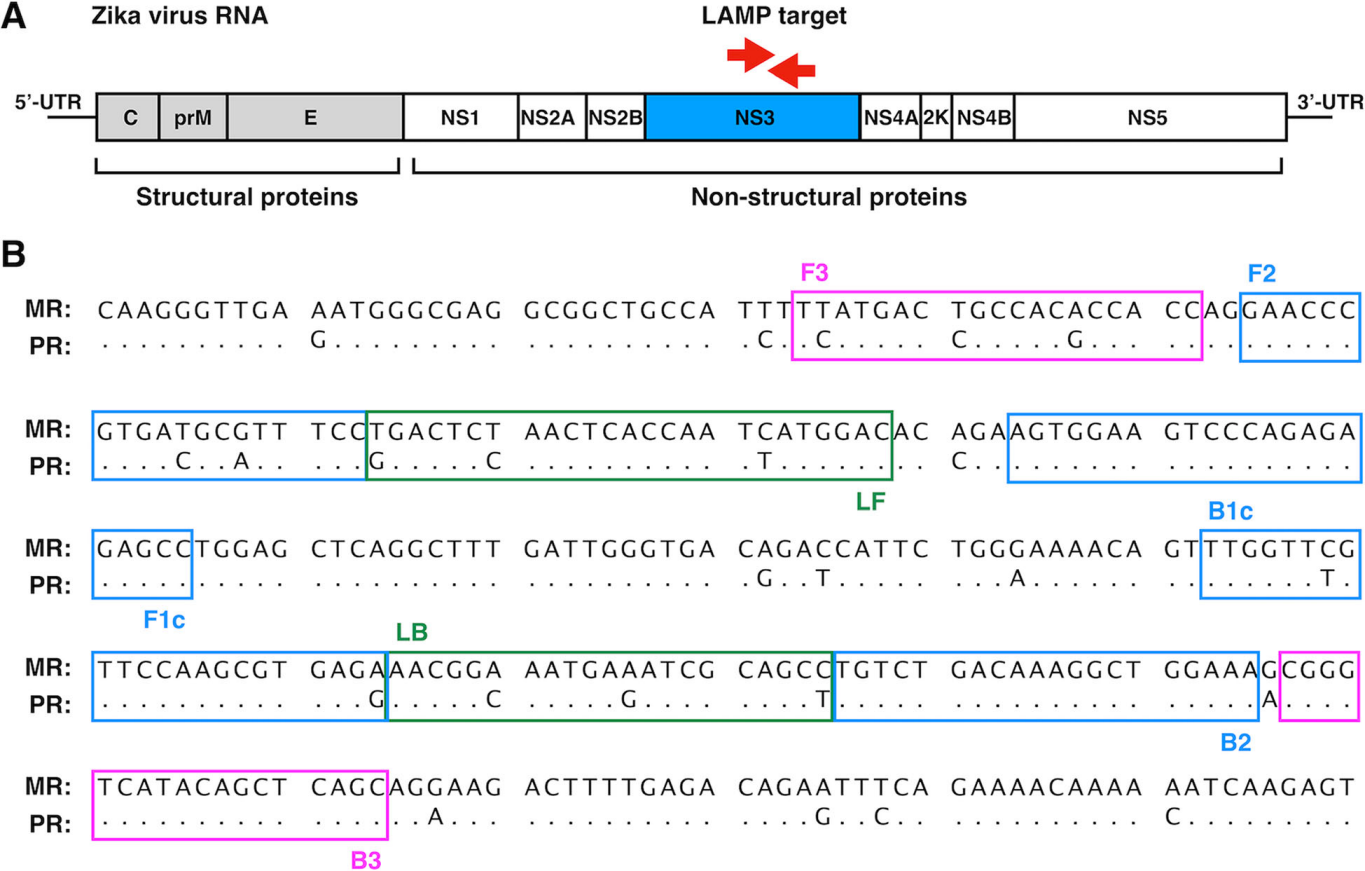
Primer set for RT-LAMP targeting ZIKV MR and PR strains. **a** Schematic diagram of the genome region of ZIKV. The region between the two red arrows indicates the target sequence of RT-LAMP. **b** Partial sequence of the ZIKV NS3 region of the MR and PR strains. The primer set was designed to amplify both target sequences of the MR and PR strains. The location of each primer, FIP (F1c-F2), BIP (B1c-B2), F3, B3, Loop-F, and Loop-B, is indicated by a box

To identify the infection of both ZIKV MR-766-NIID (MR) and PRVABC59 (PR) strains by RT-LAMP, a common primer set was designed targeting a region covering the whole ZIKV genome (Fig. 1a). Full genomic cDNA sequences of ZIKV MR-766-NIID strain (GenBank: LC002520.1) and PRVABC59 strain (GenBank: KU501215.1) were aligned, and several applicable target sequences for primer design were determined using Primer Explorer V5 (Fujitsu Ltd.), based on the nucleotide sequences of the MR strain. The primer set included several mismatches with the sequence of the PR strain (Fig. 1b). These primer sets were screened for their sensitivity in the RT-LAMP reaction for detecting genomic RNA of the ZIKV MR strain, and a primer set targeting the NS3 region of ZIKV showed the highest sensitivity (Table 1). The temperature and time for the optimized RT-LAMP reaction condition using this primer set were 62 °C and 60 min, respectively.

### Sensitivity of RT-LAMP for detecting different ZIKV strains

**Fig. 2:**
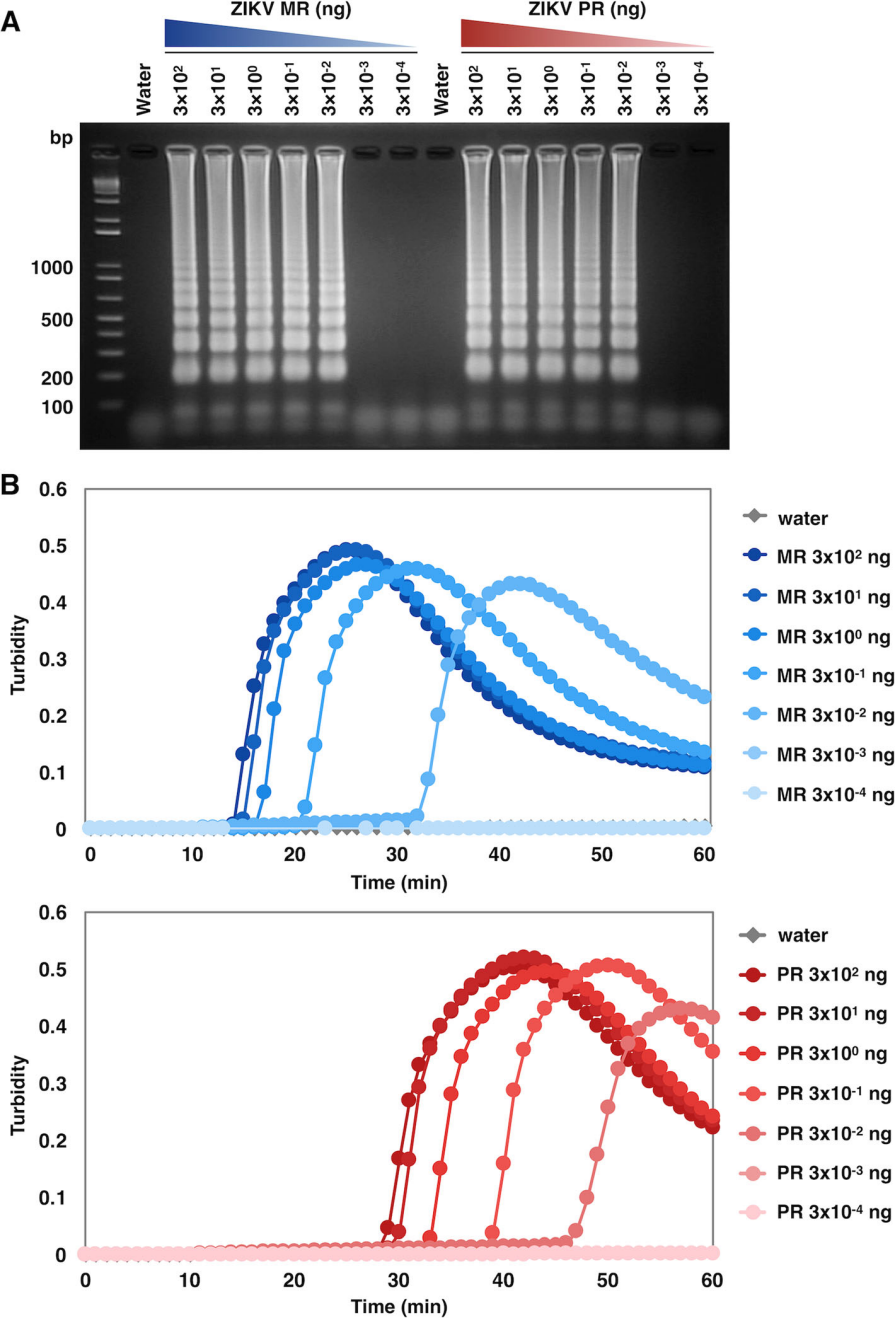
Sensitivity of RT-LAMP for the detection of ZIKV MR and PR strains. RNA from each ZIKV strain (3 × 10^2^, 3 × 10^1^, 3 × 10^0^, 3 × 10^−1^, 3 × 10^−2^, 3 × 10^−3^, and 3 × 10^−4^ ng) was used as a template for RT-LAMP reactions for 60 min at 62 °C. Water served as a negative control in RT-LAMP reaction. **a** Agarose gel electrophoresis of the RT-LAMP amplified products. The reaction mixtures were electrophoresed on a 2% agarose gel. Numbers on the left indicate migration of the molecular weight marker (bp). **b** Amplification of ZIKV NS3 of the MR and PR strains monitored using a real-time turbidimeter (turbidity at 650 nm)

To evaluate the sensitivity of ZIKV RT-LAMP using the common primer set, serially diluted ZIKV RNA was used as a template. RNAs extracted from the cell culture supernatant, which were infected with either ZIKV MR strain or PR strain, were subjected to the RT-LAMP reaction. In the optimized conditions (60 min at 62 °C), both strains were detected down to 3 × 10^−2^ ng RNA as the lower detection limit within 50 min after incubation (Fig. 2a), even when the primer set was specific to the genomic sequence of MR strain and included several mismatches to that of the PR strains (Fig. 1b). LAMP-based gene amplification can be monitored simultaneously by measuring white turbidity caused by the existence of magnesium pyrophosphate, a by-product of amplification. Real-time turbidimetry demonstrated that the primer set tended to amplify the MR strain more rapidly than the PR strain, for example, 3 × 10^2^ ng RNA of MR and PR strains became detectable as amplified product after approximately 15 min and 30 min, respectively (Fig. 2b). These results indicated high sensitivity of the newly designed primer set with optimized RT-LAMP conditions, which can be applied for discriminating among different ZIKV strains.

### ZIKV detection in clinical samples by RT-LAMP

**Fig. 3:**
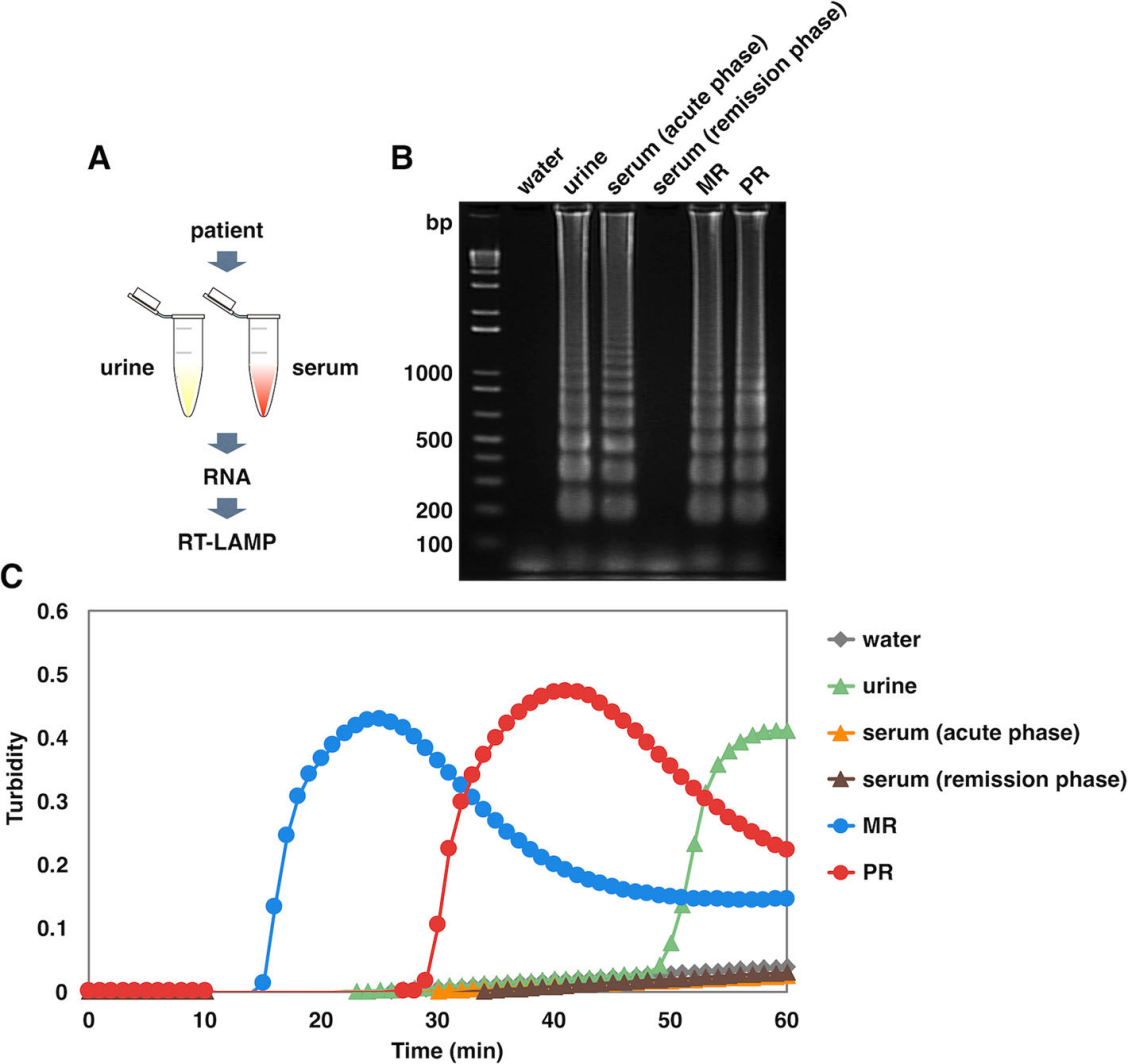
RT-LAMP-based detection of ZIKV from a Zika fever patient. **a** Scheme of preparing patient samples for RT-LAMP. RNA extracted from each patient sample (urine, serum (acute phase), and serum (remission phase)) was provided for ZIKV detection in the RT-LAMP reaction for 60 min at 62 °C. Water served as a negative control in the RT-LAMP reaction. RNA from ZIKV MR and PR served as positive controls. **b** Amplified products were electrophoresed on a 2% agarose gel. Numbers on the left indicate migration of the molecular weight marker (bp). **c** Amplification of the target sequence contained in patient samples was monitored using a real-time turbidimeter (turbidity at 650 nm)

To examine whether the ZIKV RT-LAMP can disclose the existence of viral RNA in human samples that usually contain a variety of debris such as nucleotides in host cells, urine and serum from a ZIKV-infected patient were tested. A middle-aged male who visited the Dominican Republic in 2016 was diagnosed with Zika fever, and the patient’s serum and urine were collected in both acute and remission phase of the disease; the initial and following sera were collected at the first visit of the patient with remarkable symptoms and at the second visit when symptoms were mitigated. Urine was also collected from the same patient in remission. RNA was extracted from each sample and then subjected to the RT-LAMP reaction (Fig. 3a). It resulted in detecting ZIKV in both urine and serum (acute phase), but not in serum (remission phase), by gel electrophoresis (Fig. 3b). The turbidimetry analysis also identified amplified products from the urine sample but not from serum samples presumably due to low-level viremia below the detection limit (Fig. 3c). These results demonstrated that the ZIKV RT-LAMP is applicable for the potential diagnosis of Zika fever using urine and serum.

### Possible estimation of ZIKV lineage by RT-LAMP

**Fig. 4:**
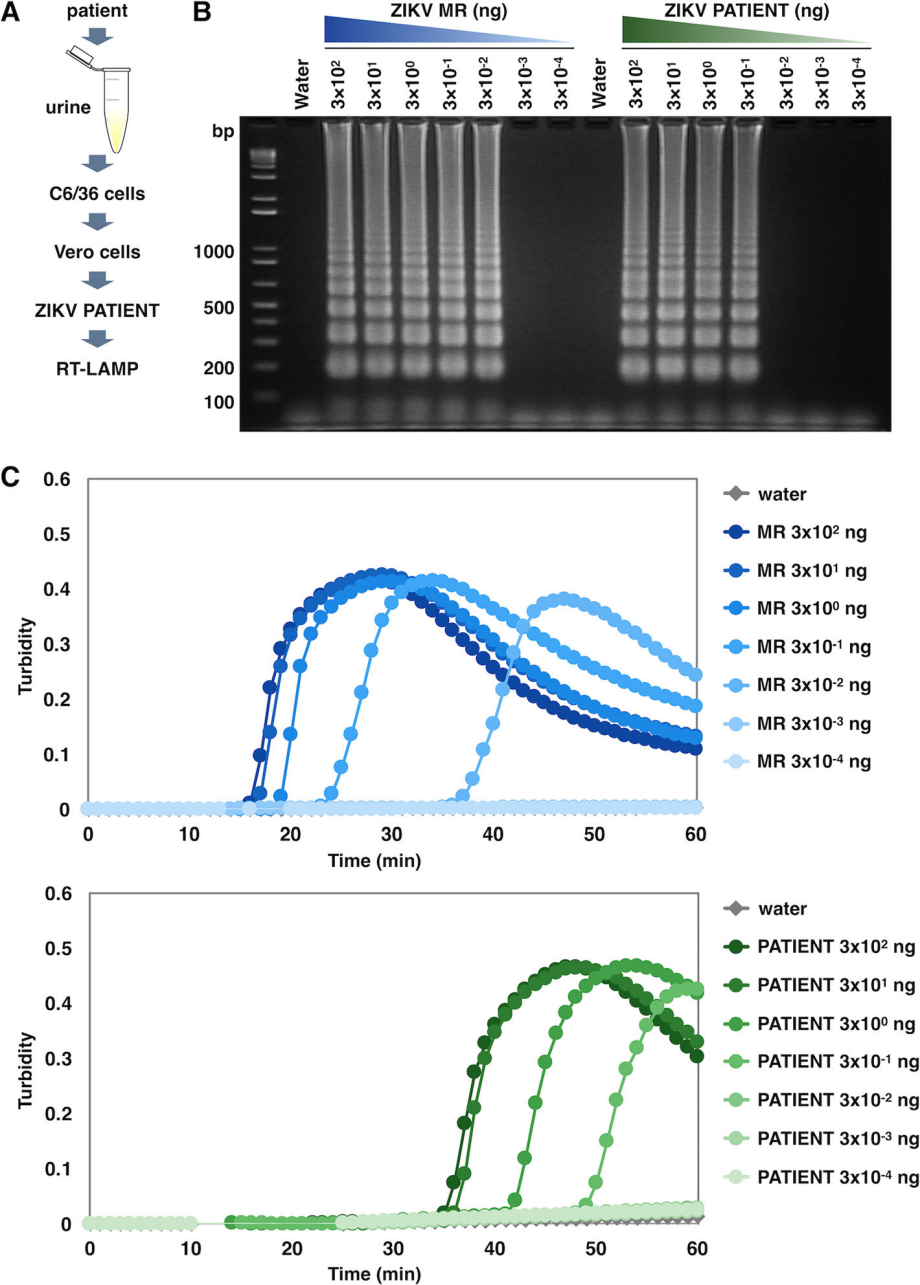
Sensitivity of RT-LAMP for the detection of ZIKV from Zika fever patient. **a** Scheme for preparing the ZIKV PATIENT strain from patient’s urine for RT-LAMP detection. **b**, **c** Amplification of ZIKV NS3 from the MR and PATIENT strains by RT-LAMP. RNA of each ZIKV strain (3 × 10^2^, 3 × 10^1^, 3 × 10^0^, 3 × 10^−1^, 3 × 10^−2^, 3 × 10^−3^, and 3 × 10^−4^ ng) was used as a template for RT-LAMP reactions for 60 min at 62 °C. Water served as a negative control in the RT-LAMP reaction. **b** Agarose gel electrophoresis of the RT-LAMP amplified products. The reaction mixtures were electrophoresed on a 2% agarose gel. Numbers of the left indicate migration of the molecular weight marker (bp). **c** Amplification of the target sequence of the MR and PATIENT strains was monitored using a real-time turbidimeter (turbidity at 650 nm)

To explore where the ZIKV RT-LAMP reaction can provide information about unidentified ZIKV strains, the virus observed in the Zika fever patient (as shown in Fig. 3) was isolated. At first, cultured cells were spiked with the patient’s urine for propagation of ZIKV, which was designated as ZIKV PATIENT. Viral RNA was then extracted from the ZIKV PATIENT strain, and the RT-LAMP reaction was performed in optimized conditions (Fig. 4a). RNA of the PATIENT strain less than 3 × 10^−2^ ng failed to be detected by either turbidimetry or gel electrophoresis, compared with MR strain (Fig. 4b, c). Turbidimetry observed in the RT-LAMP reaction amplified the PATIENT strain more slowly than the MR strain; 3 × 10^2^ ng RNA of MR and PATIENT strain became detectable as amplified product after approximately 15 min and 35 min, respectively (Fig. 4c).

**Fig. 5:**
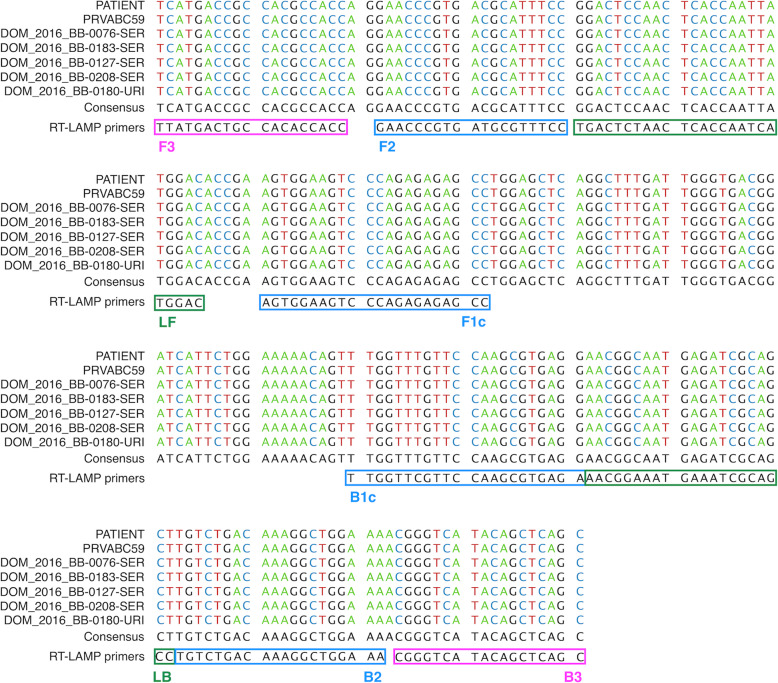
Multiple sequence alignment of ZIKV PATIENT with PR and previously reported ZIKV isolated in the Dominican Republic. RT-LAMP primers, which are specific for MR, are indicated by boxes

The partial cDNA sequence of the PATIENT strain, which contained the target region for RT-LAMP, was determined and aligned with the sequences of five ZIKV strains isolated in the Dominican Republic. The sequence of the PATIENT strain completely matched the PR strain and all five Dominican strains (Fig. 5), suggesting that the PATIENT strain belonged to the Asian lineage. The result was also consistent with the observed lag in reaction times for amplification among the MR, PR, and PATIENT strains (Figs. 2b and 4c). The findings of this study suggested that the RT-LAMP method with a common primer set may be a feasible tool not only for detecting ZIKV with high sensitivity but also for estimating the specific lineage of ZIKV.

## Discussion

After a large outbreak of Zika virus disease in 2015, faster and more accurate diagnostic methods have been widely sought for risk assessment. So far, serological testing to detect antibodies and nucleic acid testing, including RT-PCR, to detect target sequences in the virus genome have been used as standard diagnostic methods [18].

In this present study, we demonstrated that ZIKV in patient’s blood serum and urine was detectable by RT-LAMP with a common primer set designed to detect two ZIKV standard strains, MR-766-NIID and PRVABC59. Although the RT-LAMP method efficiently detected both ZIKV strains, the sensitivity for each strain clearly differed, which was observed by measuring the reaction times necessary for turbidity detection. This can be explained by the sequence of each primer, which was designed to match prior to the sequence of the MR-766-NIID strain. The amplification of ZIKV sequence from the patient samples by RT-LAMP was similar to that of PRVABC59, suggesting similar variation of viral genome sequence to PRVABC59 rather than that of MR-766-NIID. Detection of ZIKV by RT-LAMP has been reported previously, in which several standard strains were used as templates [14, 15]. Our findings, detecting both laboratory standard strains and clinical strains by RT-LAMP, support those works and suggest the importance of consideration about variations in genome sequences in the use of RT-LAMP with a common primer set for ZIKV detection.

The RT-LAMP method developed in this study detected ZIKV in urine and blood serum collected at the first visit of the patient to the hospital, but not in blood serum at the second visit. This finding suggested that viral RNA had disappeared from the blood serum between the first and second visits, in agreement with the amelioration of symptoms of the patient. The Centers for Disease Control and Prevention (CDC) has so far recommended testing by RT-PCR with serum within the first 7 days of illness onset, and with urine collected less than 14 days after symptoms onset [26, 27]. Because the symptom onset of the patient was not clear, the day of the second visit may have been later than the virus RNA-detectable period in serum. Examining the patient’s urine at each time point in addition to serum by RT-LAMP may be necessary for accurate diagnosis of Zika virus disease.

The lower detection limit of the PATIENT strain was 3 × 10^−1^ ng RNA, which was higher than that of the PR strain (3 × 10^−2^ ng), even the target sequences of PR and PATIENT strains were identical. This may be explained by the presence of non-target RNAs in the sample; the sample of PATIENT strain may contain a large amount of RNA derived from patient cells and urine compared to viral RNA. Additionally, it is known that delayed LAMP reaction with lower sensitivity is occasionally observed when sample contains non-target nucleotides. Although we tested serially diluted samples based on the weight of purified RNA, further assessment using samples specified for virus copies or infectious units will provide more adequate information for diagnostic use.

RT-LAMP employs four to six primers targeting six to eight regions of a gene, allowing high specificity. The RT-LAMP method described in this study demonstrated no amplification of dengue virus 2 (DENV-2) sequence (manuscript in preparation). The reported NS3 sequences of flaviviruses, including DENV-1-4, West Nile, and yellow fever virus, suggest that their sequences are unlikely amplified by this RT-LAMP due to mismatches in the primer regions critical for DNA extension [4]. When applying the RT-LAMP method in ZIKV endemic areas, further experiments are necessary to confirm for no false amplification against other flaviviruses common in the areas.

## Conclusions

The present study showed that the RT-LAMP method can achieve high specificity and sensitivity for detecting multiple strains of ZIKV. Implementing RT-LAMP with a carefully selected common primer set may be a promising strategy for the diagnosis of patients with ZIKV infection.

## Data Availability

The datasets supporting the conclusion of this study are included within the article.
